# Supplementary material for: Comparison between Intramuscular and Intranasal Administration of Sedative Drugs Used for Piglet Castration
Source: Animals (Basel). 2024 Aug 12;14(16):2325. doi: 10.3390/ani14162325 (PMC11350754; doi:10.3390/ani14162325)
Supplement: Supplementary file 1 [file animals-14-02325-s001.zip › animals-3118168-supplementary.pdf]

Supplementary Materials:

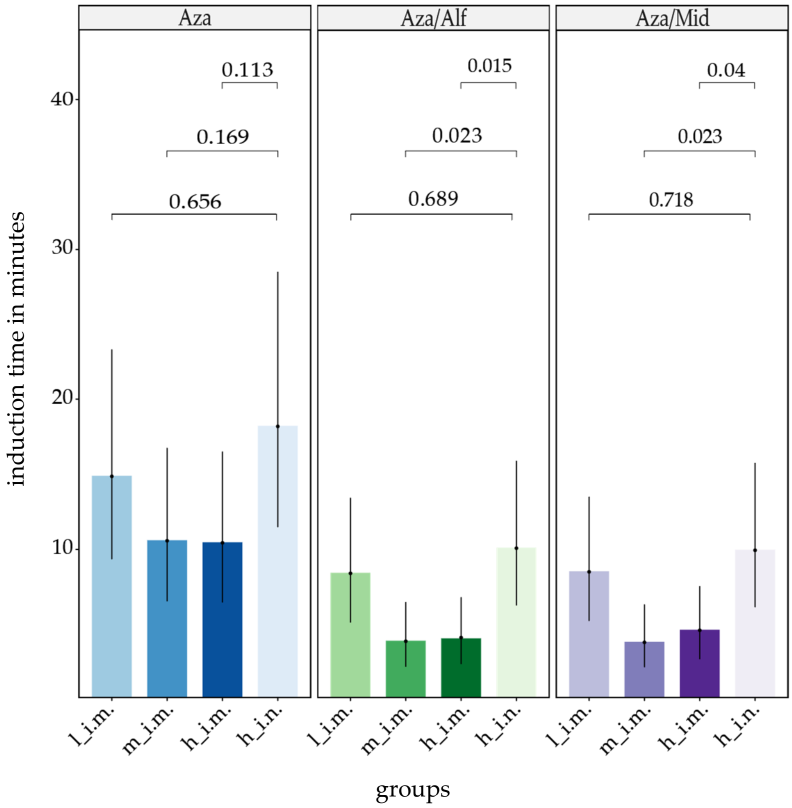

Supplementary figure S1: Estimated marginal means of induction times after sedation with Azaperone 5mg/kg i.n. (Aza_h_i.n.), Azaperone 5mg/kg i.m. (Aza_h_i.m.), Azaperone 3mg/kg i.m. (Aza_m_i.m.), Azaperone 2mg/kg i.m. (Aza_l_i.m.), Azaperone 5mg/kg + Alfaxalone 5mg/kg i.n. (Aza/Alf_h_i.n.), Azaperone 5mg/kg + Alfaxalone 5mg/kg i.m. (Aza/Alf_h_i.m.), Azaperone 3mg/kg + Alfaxalone 3mg/kg i.m. (Aza/Alf_m_i.m.), Azaperone 2mg/kg + Alfaxalone 2mg/kg i.m. (Aza/Alf_l_i.m.), Azaperone 5mg/kg + Midazolam 0.2mg/kg i.n. (Aza/Mid_h_i.n.), Azaperone 5mg/kg + Midazolam 0.2mg/kg i.m. (Aza/Mid_h_i.m.), Azaperone 3mg/kg + Midazolam 0.2mg/kg i.m. (Aza/Mid_m_i.n.) and Azaperone 2mg/kg + Midazolam 0.2mg/kg i.m. (Aza/Mid_l_i.n.)

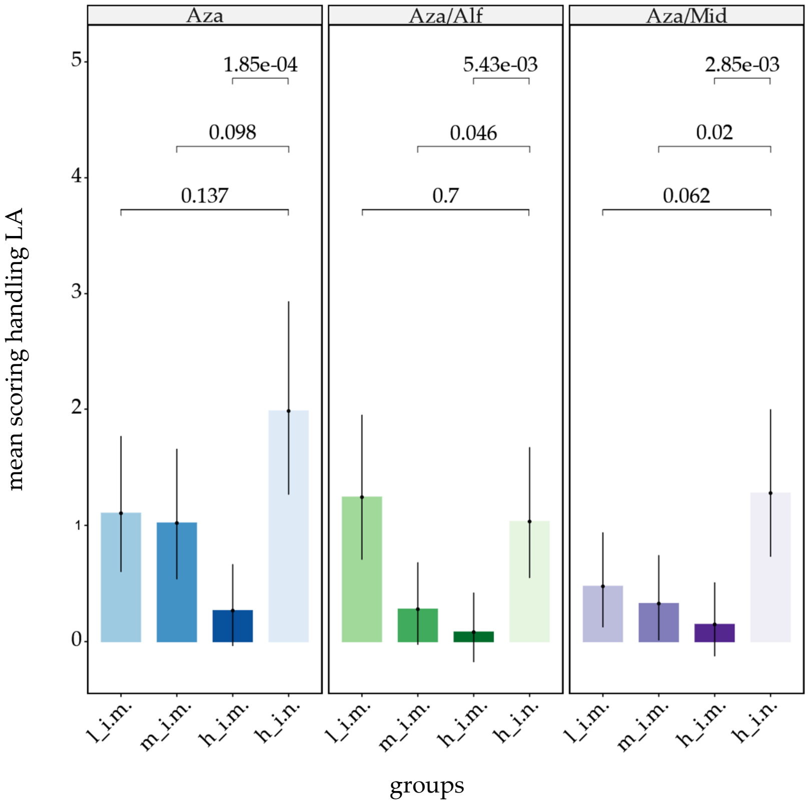

Supplementary figure S2: Estimated marginal means of scores of movement and vocalisation throughout handling and fixation for application of local anaesthetics (LA) after sedation with Azaperone 5mg/kg i.n. (Aza_h_i.n.), Azaperone 5mg/kg i.m. (Aza_h_i.m.), Azaperone 3mg/kg i.m. (Aza_m_i.m.), Azaperone 2mg/kg i.m. (Aza_l_i.m.), Azaperone 5mg/kg + Alfaxalone 5mg/kg i.n. (Aza/Alf_h_i.n.), Azaperone 5mg/kg + Alfaxalone 5mg/kg i.m. (Aza/Alf_h_i.m.), Azaperone 3mg/kg + Alfaxalone 3mg/kg i.m. (Aza/Alf_m_i.m.), Azaperone 2mg/kg + Alfaxalone 2mg/kg i.m. (Aza/Alf_l_i.m.), Azaperone 5mg/kg + Midazolam 0.2mg/kg i.n. (Aza/Mid_h_i.n.), Azaperone 5mg/kg + Midazolam 0.2mg/kg i.m. (Aza/Mid_h_i.m.), Azaperone 3mg/kg + Midazolam 0.2mg/kg i.m. (Aza/Mid_m_i.n.) and Azaperone 2mg/kg + Midazolam 0.2mg/kg i.m. (Aza/Mid_l_i.n.)

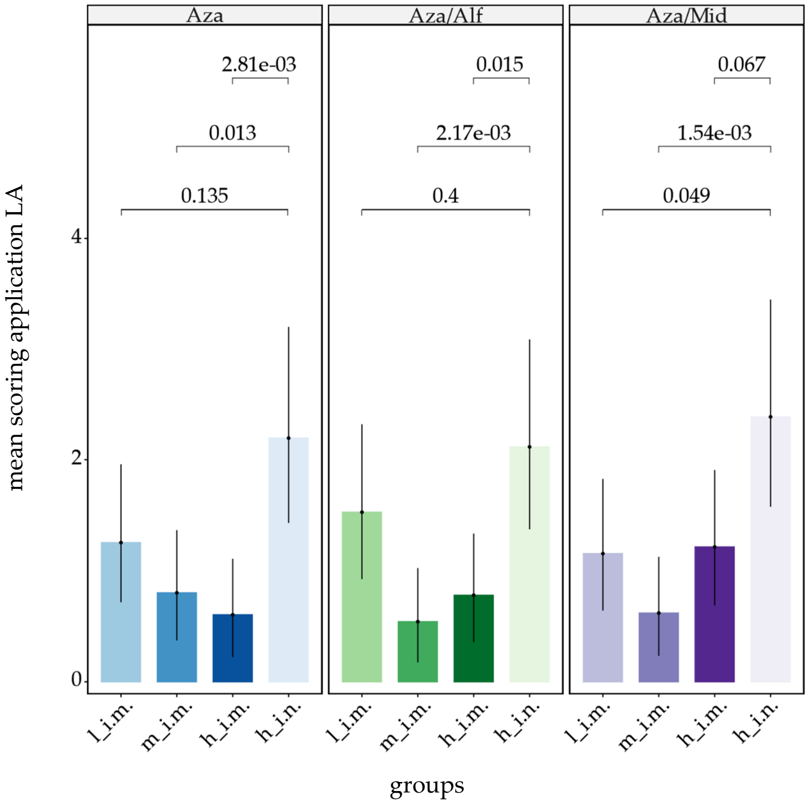

Supplementary figure S3: Estimated marginal means of scores of movement and vocalisation throughout application of local anaesthetics (LA) after sedation with Azaperone 5mg/kg i.n. (Aza_h_i.n.), Azaperone 5mg/kg i.m. (Aza_h_i.m.), Azaperone 3mg/kg i.m. (Aza_m_i.m.), Azaperone 2mg/kg i.m. (Aza_l_i.m.), Azaperone 5mg/kg + Alfaxalone 5mg/kg i.n. (Aza/Alf_h_i.n.), Azaperone 5mg/kg + Alfaxalone 5mg/kg i.m. (Aza/Alf_h_i.m.), Azaperone 3mg/kg + Alfaxalone 3mg/kg i.m. (Aza/Alf_m_i.m.), Azaperone 2mg/kg + Alfaxalone 2mg/kg i.m. (Aza/Alf_l_i.m.), Azaperone 5mg/kg + Midazolam 0.2mg/kg i.n. (Aza/Mid_h_i.n.), Azaperone 5mg/kg + Midazolam 0.2mg/kg i.m. (Aza/Mid_h_i.m.), Azaperone 3mg/kg + Midazolam 0.2mg/kg i.m. (Aza/Mid_m_i.n.) and Azaperone 2mg/kg + Midazolam 0.2mg/kg i.m. (Aza/Mid_l_i.n.)

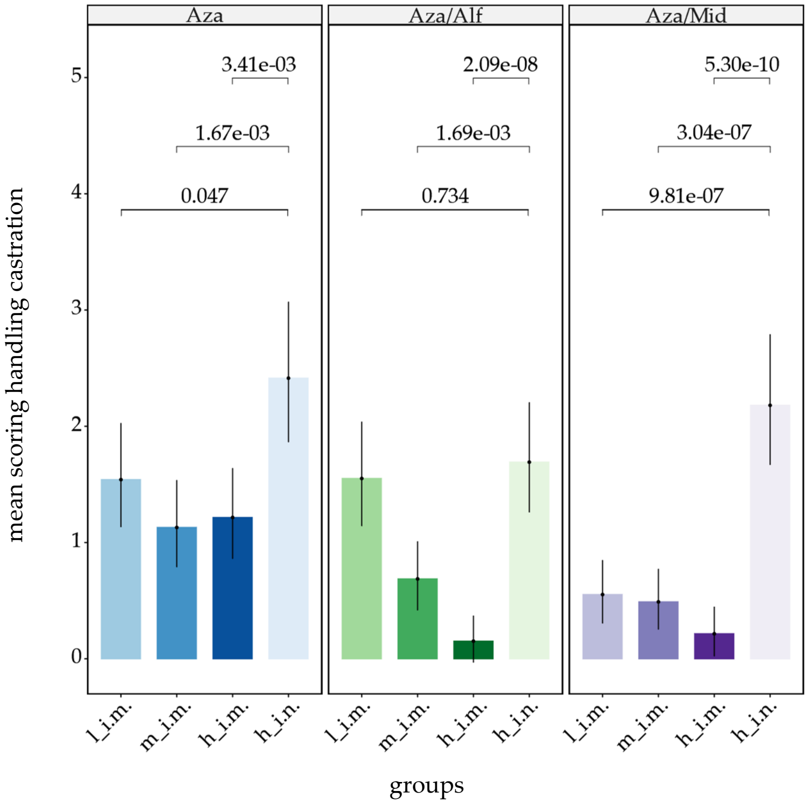

Supplementary figure S4: Estimated marginal means of scores of movement and vocalisation throughout handling and fixation for castration after sedation with Azaperone 5mg/kg i.n. (Aza_h_i.n.), Azaperone 5mg/kg i.m. (Aza_h_i.m.), Azaperone 3mg/kg i.m. (Aza_m_i.m.), Azaperone 2mg/kg i.m. (Aza_l_i.m.), Azaperone 5mg/kg + Alfaxalone 5mg/kg i.n. (Aza/Alf_h_i.n.), Azaperone 5mg/kg + Alfaxalone 5mg/kg i.m. (Aza/Alf_h_i.m.), Azaperone 3mg/kg + Alfaxalone 3mg/kg i.m. (Aza/Alf_m_i.m.), Azaperone 2mg/kg + Alfaxalone 2mg/kg i.m. (Aza/Alf_l_i.m.), Azaperone 5mg/kg + Midazolam 0.2mg/kg i.n. (Aza/Mid_h_i.n.), Azaperone 5mg/kg + Midazolam 0.2mg/kg i.m. (Aza/Mid_h_i.m.), Azaperone 3mg/kg + Midazolam 0.2mg/kg i.m. (Aza/Mid_m_i.n.) and Azaperone 2mg/kg + Midazolam 0.2mg/kg i.m. (Aza/Mid_l_i.n.)

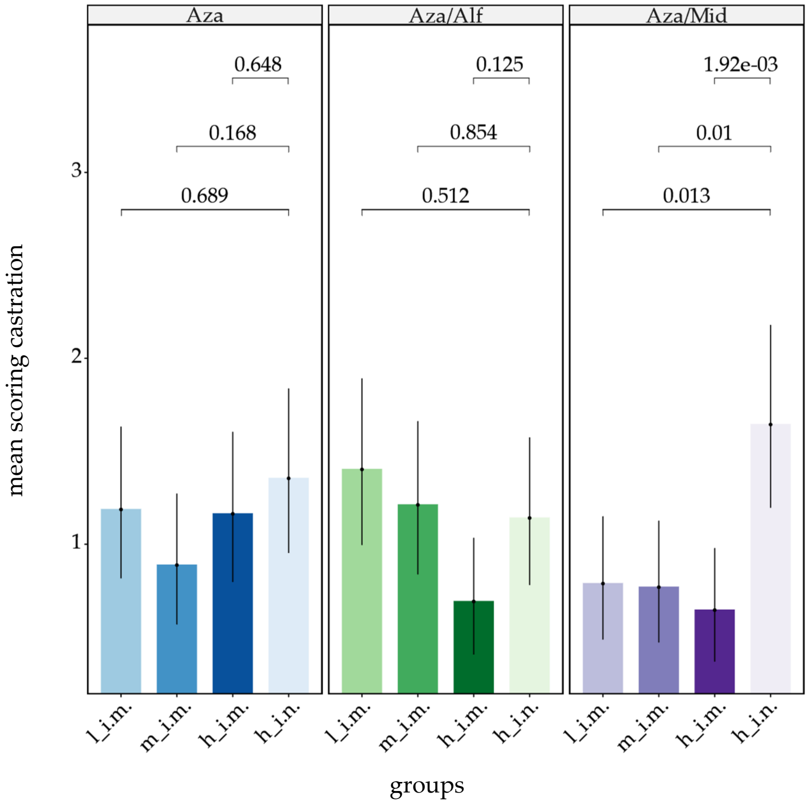

Supplementary figure S5: Estimated marginal means of scores of movement and vocalisation throughout castration after sedation with Azaperone 5mg/kg i.n. (Aza_h_i.n.), Azaperone 5mg/kg i.m. (Aza_h_i.m.), Azaperone 3mg/kg i.m. (Aza_m_i.m.), Azaperone 2mg/kg i.m. (Aza_l_i.m.), Azaperone 5mg/kg + Alfaxalone 5mg/kg i.n. (Aza/Alf_h_i.n.), Azaperone 5mg/kg + Alfaxalone 5mg/kg i.m. (Aza/Alf_h_i.m.), Azaperone 3mg/kg + Alfaxalone 3mg/kg i.m. (Aza/Alf_m_i.m.), Azaperone 2mg/kg + Alfaxalone 2mg/kg i.m. (Aza/Alf_l_i.m.), Azaperone 5mg/kg + Midazolam 0.2mg/kg i.n. (Aza/Mid_h_i.n.), Azaperone 5mg/kg + Midazolam 0.2mg/kg i.m. (Aza/Mid_h_i.m.), Azaperone 3mg/kg + Midazolam 0.2mg/kg i.m. (Aza/Mid_m_i.n.) and Azaperone 2mg/kg + Midazolam 0.2mg/kg i.m. (Aza/Mid_l_i.n.)

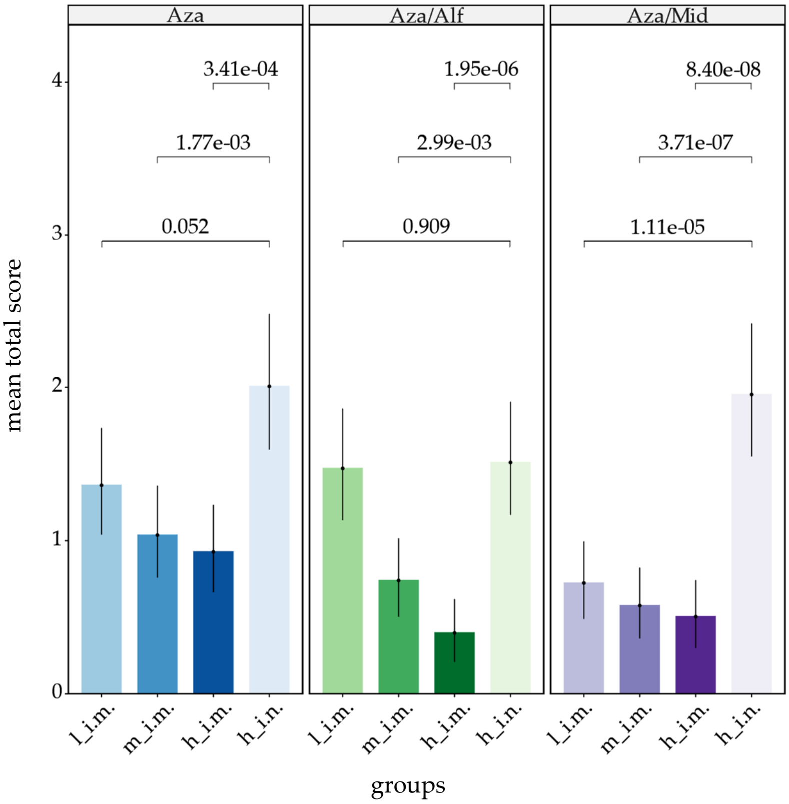

Supplementary figure S6: Estimated marginal means of scores of all assessed steps after sedation with Azaperone 5mg/kg i.n. (Aza_h_i.n.), Azaperone 5mg/kg i.m. (Aza_h_i.m.), Azaperone 3mg/kg i.m. (Aza_m_i.m.), Azaperone 2mg/kg i.m. (Aza_l_i.m.), Azaperone 5mg/kg + Alfaxalone 5mg/kg i.n. (Aza/Alf_h_i.n.), Azaperone 5mg/kg + Alfaxalone 5mg/kg i.m. (Aza/Alf_h_i.m.), Azaperone 3mg/kg + Alfaxalone 3mg/kg i.m. (Aza/Alf_m_i.m.), Azaperone 2mg/kg + Alfaxalone 2mg/kg i.m. (Aza/Alf_l_i.m.), Azaperone 5mg/kg + Midazolam 0.2mg/kg i.n. (Aza/Mid_h_i.n.), Azaperone 5mg/kg + Midazolam 0.2mg/kg i.m. (Aza/Mid_h_i.m.), Azaperone 3mg/kg + Midazolam 0.2mg/kg i.m. (Aza/Mid_m_i.n.) and Azaperone 2mg/kg + Midazolam 0.2mg/kg i.m. (Aza/Mid_l_i.n.)

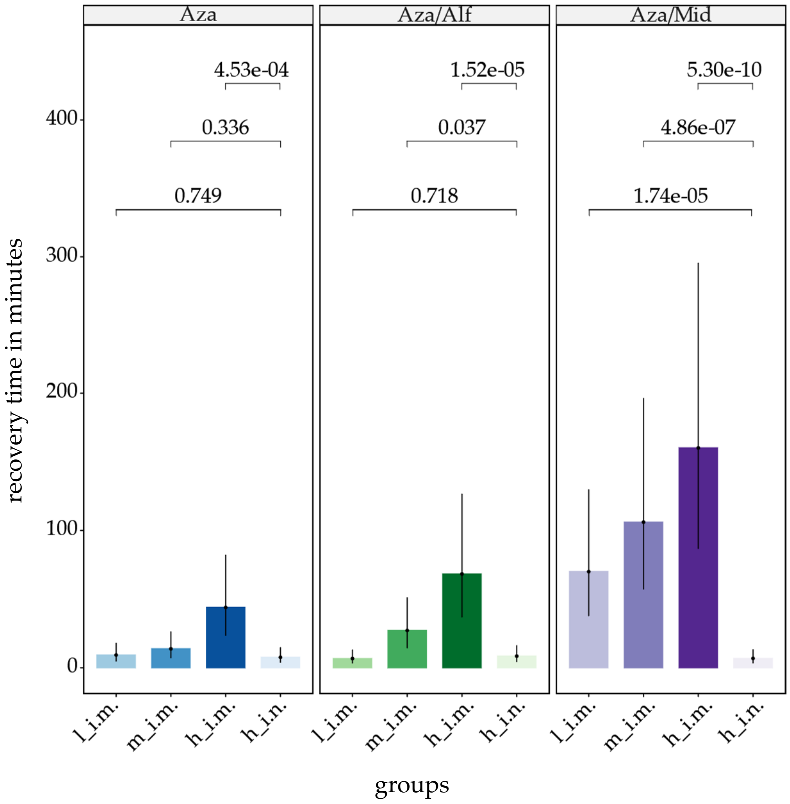

Supplementary figure S7: Estimated marginal means of recovery times after castration following sedation with Azaperone 5mg/kg i.n. (Aza_h_i.n.), Azaperone 5mg/kg i.m. (Aza_h_i.m.), Azaperone 3mg/kg i.m. (Aza_m_i.m.), Azaperone 2mg/kg i.m. (Aza_l_i.m.), Azaperone 5mg/kg + Alfaxalone 5mg/kg i.n. (Aza/Alf_h_i.n.), Azaperone 5mg/kg + Alfaxalone 5mg/kg i.m. (Aza/Alf_h_i.m.), Azaperone 3mg/kg + Alfaxalone 3mg/kg i.m. (Aza/Alf_m_i.m.), Azaperone 2mg/kg + Alfaxalone 2mg/kg i.m. (Aza/Alf_l_i.m.), Azaperone 5mg/kg + Midazolam 0.2mg/kg i.n. (Aza/Mid_h_i.n.), Azaperone 5mg/kg + Midazolam 0.2mg/kg i.m. (Aza/Mid_h_i.m.), Azaperone 3mg/kg + Midazolam 0.2mg/kg i.m. (Aza/Mid_m_i.n.) and Azaperone 2mg/kg + Midazolam 0.2mg/kg i.m. (Aza/Mid_l_i.n.)
